# Immune Thrombocytopenic Purpura Secondary to Cytomegalovirus Infection: A Case Report

**DOI:** 10.3389/fmed.2015.00079

**Published:** 2015-11-03

**Authors:** Bessy S. Flores-Chang, Carlos E. Arias-Morales, Francis G. Wadskier, Sorab Gupta, Nicoleta Stoicea

**Affiliations:** ^1^Department of Internal Medicine, St. Barnabas Hospital Bronx, Bronx, NY, USA; ^2^Department of Anesthesiology, The Ohio State University Wexner Medical Center, Columbus, OH, USA

**Keywords:** immune thrombocytopenic purpura, cytomegalovirus, steroids, valgancyclovir, bleeding

## Abstract

Immune thrombocytopenic purpura (ITP) is defined as an acquired thrombocytopenia with antibodies detected against platelet surface antigens, and it is the most common form of thrombocytopenia in otherwise asymptomatic adults. ITP secondary to an underlying condition is a diagnosis of exclusion that is essential to establish for treatment efficacy. Secondary thrombocytopenia caused by cytomegalovirus (CMV) is common; however, case reports associated with diagnosis in immunocompetent adults are rare, and to the best of our knowledge only 20 publications have been associated with this diagnosis. Our report is based on a clinical presentation of a 37-year-old female complaining of petechiae, heavy menses, shortness of breath, and a platelet count of 1 × 10^9^/L. Treatment with IVIG and steroids failed to improve platelet count. Subsequently, an infectious laboratory workup was performed, detecting CMV infection, and treatment with antiviral agents was initiated, causing platelet count to increase as viral load decreased.

## Introduction

Immune thrombocytopenic purpura (ITP) is a form of acquired thrombocytopenia triggered by anti-platelet antibodies that destroy platelets peripherally, damage megakaryocytes, and inhibits platelet production in the marrow (1). Agents such as CMV, hepatitis C, Epstein–Barr virus, and Parvovirus B19, among others, have been identified as the culprits behind this form of ITP (2). Steroids and IVIG are the treatment of choice for ITP. The primary infection should be treated in order to allow ITP standard therapies to be effective. We present a case of a 37-year-old female patient with a history of petechia, heavy menstrual bleed, and low platelets (1 × 10^9^/L) after a CMV infection. Cytomegalovirus (CMV) is a known cause of morbidity and mortality in patients with immunosuppressed states, whereas in the immunocompetent patients, the virus commonly manifests as an asymptomatic infection or as mononucleosis-like syndrome. CMV can cause disease in immunocompromised patients either via a primary CMV infection or reactivation of a latent CMV infection. It is characterized by malaise, myalgia, headache, sore throat, and fever. Associated clinical syndromes include encephalitis, pneumonitis, hepatitis, uveitis, retinitis, colitis, and graft rejection. Treatment of the primary infection is imperative because standard therapies for ITP regain their efficacy once the infection is resolved (3).

## Case Presentation

**Figure 1:**
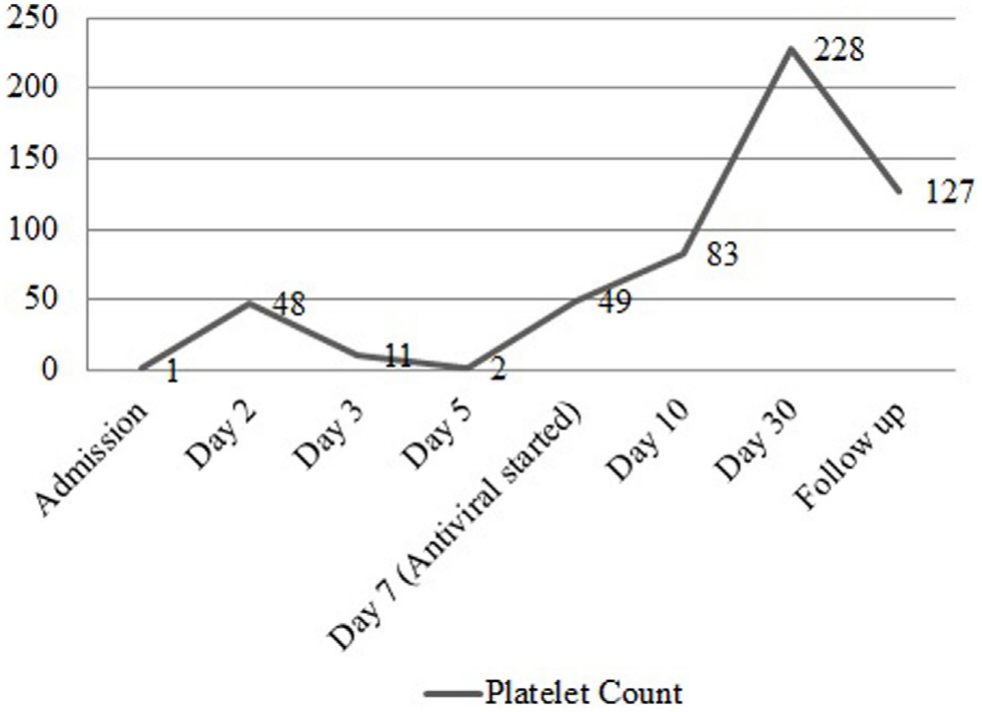
**Platelet count before and after treatment**. Graph representing the trend of platelet count from the time of admission to the post treatment period with valgancyclovir, romiplostim, and IVIG.

**Figure 2:**
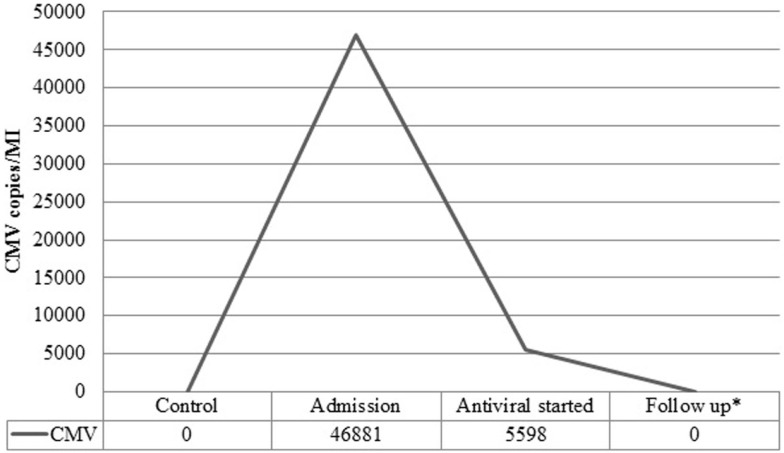
**Viral load before and after treatment**. Viral load in copies/MI for Cytomegalovirus (CMV) measured at admission, post treatment with valgancyclovir and during follow-up.

A 37-year-old female presented to the emergency department complaining of shortness of breath, pruritic rash, epistaxis, heavy menses, and night sweats. A physical examination revealed a petechial rash on her torso, lower extremities, and oral mucosa. On admission, the patient was found to have platelet count of 1.0 × 10^9^/L (Figure 1).The normal range for platelets in healthy Caucasians is 150,000–400,000/mm^3^ (a cubic millimeter equals a microliter), or 150–400 × 10^9^/L (4). The patient’s vitals were stable, including the absence of fever, and on physical examination no splenomegaly was found. White blood cells (WBC) and red blood cells (RBC) were unremarkable (Table 1); nonetheless, a slight elevation in AST and ALT was found. The patient’s symptoms aroused suspicion of immune thrombocytopenia. Initial administration of high-dose steroids (dexamethasone 40 mg PO ×4 days and prednisone 60 mg PO ×2 days) showed improvement in the patient’s platelet count; however a subsequent course of high-dose methylprednisolone (solumedrol 60 mg IV ×3 days) did not show any further clinical benefit. Intravenous Immunoglobulin (IVIG 1 g/kg ×2 days) was administered, and far from alleviating existing symptoms, caused right pleural effusion, prompting its discontinuation and the possibility of secondary ITP was suspected. An infectious work up was performed, and results were all negative except by PCR for CMV–DNA that showed CMV IgM more than fourfold, a viral load of 46,881 copies/MI (Figure 2). A bone marrow biopsy revealed a combined pattern of normocellular and hypercellular areas along with the presence of megakaryocytes. Per Infectious Disease and Hematology-Oncology medical team recommendations, a combination of valgancyclovir (900 mg PO BID) and romiplostin (Nplate^®^ 1 μg/kg SC/week) was started, causing a significant drop in viral load (Figure 2). Resolution of CMV infection and subsequent promotion of platelet growth contributed to a significant improvement in patient’s symptoms. The patient was discharged with a platelet count of 49 × 10^9^/L (Figure 1). One month later, platelet count improved to 228 × 10^9^/L, and CMV viral load dropped to <200 copies/MI and asymptomatic (Figure 2).

**Table 1:** **Complete blood count (CBC) with differential during the course of the disease**.

	Admission	Day 2	Day 3	Day 5	Day 10	Day 30	3 months Follow-up
WBC (10^3^/μL)	5.6	9.4	6.6	9.2	9	10.6	8.7
RBC (10^6^/μL)	4.41	3.92	3.55	3.25	3.27	3.94	4.18
Hgb (g/dL)	13.7	12.1	10.7	10.4	10	11.8	12.3
Hct (%)	40.9	35.8	32.9	32	30.6	37.7	39.6
RDW (%)	12.7	12.8	12.9	13.3	13.5	14.4	14.9
Neutrophil (%)	42	77.4	74.2	65.6	71.9	73.5	55.8
Lymphocyte (%)	46	19	21.5	23.4	15	20.4	30.8
Monocyte (%)	8.4	1.4	3.5	7.8	10.9	4.1	11.2
Eosinophil (%)	1.1	0	0	0	0	0.2	0.3
Basophil (%)	1.4	0.5	0.2	0.5	0.4	0.2	0.3
Immature granulocyte (%)	1.1	1.7	1.2	2.7	1.8	2	1.6

## Informed Consent Statement

The subject described in this report has given verbal informed consent. Written informed consent is not required if data is drawn from observed behavior or if data does not contain identifying information, as it is in the present report.

## Discussion

Secondary ITP is an acquired thrombocytopenia caused by autoantibodies against platelets (5). Initial presentations of ITP are petechiae and purpura, with a more severe progression to intracranial hemorrhage or gastrointestinal bleeding, leading to a fatal outcome, if treatment is not started on a promptly manner (3, 5). Chronic conditions, such as systemic lupus erythematosus (SLE), chronic lymphocytic leukemia (CLL), and infectious agents such as immunodeficiency virus (HIV), hepatitis C virus (HCV), and CMV, can trigger this form of ITP (5–7). The various theories of CMV infection-induced thrombocytopenia were described by Crapnell et al., as CMV-induced direct cytotoxicity to hematopoietic cells with immune-mediated destruction of infected cells or impairment of bone marrow stromal function (7).

Immune thrombocytopenic purpura is frequently an exclusionary diagnosis, characterized by the absence of any other clinical condition accountable for the low platelet count and isolated thrombocytopenia. Generally, risk of bleeding in ITP is low, but it increases when platelet count is <10 × 10^9^/L. Initial treatment should focus on preventing clinically significant bleeding and treating the primary infection, rather than to normalize platelet count, as the risk of bleeding is low even in patients with severely low platelet counts (5). Some physicians believe treatment with steroids should be avoided in patients with CMV-induced thrombocytopenia, as immunosuppressive treatment may be responsible for the primary CMV infection exacerbation and trigger further decrease in platelet count (3, 8). It has been demonstrated that standard therapies for ITP, including IVIG and splenectomy, regain their efficacy once the primary CMV infection is controlled with antiviral agents such as ganciclovir or valganciclovir (3, 9). Treatment with ganciclovir or valganciclovir is currently recommended as first-line treatment for immunecompromised adults with severe CMV disease, and few studies have evaluated the use of these antiviral drugs for treatment in immunocompetent adults due to the major side effects of these agents such as myelosupression (10). Nevertheless, untreated CMV disease in immunocompetent adults is associated with increased morbidity and mortality. There are some studies that provide evidence of significant clinical improvement after treatment with antiviral agents is started (10–13). On the other hand, the use of thrombopoeitin receptor (TPO-R) agonists such as romiplostin (Nplate^®^) has been studied, and literature has shown that these agents are effective in refractory ITP compared to placebo (14–16).

## Conclusion

Our case report will add to the existing body of research and will increase awareness of this form of thrombocytopenia. Further research is needed in order to establish general treatment guidelines for CMV-induced thrombocytopenia in immunocompetent patients while steroids administration is still debatable.

## Conflict of Interest Statement

The authors declare that the research was conducted in the absence of any commercial or financial relationships that could be construed as a potential conflict of interest.
